# Effect of Stress and Caffeine on Male Infertility

**DOI:** 10.7759/cureus.28487

**Published:** 2022-08-27

**Authors:** Mayank Kumar, Sarju Zilate, Chirag Gupta

**Affiliations:** 1 Pharmacology, Jawaharlal Nehru Medical College, Datta Meghe Institute of Medical Sciences, Wardha, IND

**Keywords:** caffeine, stress, male infertility, coffee, caffeinated drinks, male fertility

## Abstract

Caffeine is a natural psychoactive chemical found in beverages made from coffee. In addition, it is added by the manufacturers of a large number of sodas and energy drinks. It does this by stimulating both the brain and the central nervous system, enabling you to avoid being sleepy while still keeping you attentive. A state of emotional or physical tension is defined as stress. It can be induced through the experience of something or thinking of something that causes you to feel uncomfortable, irritated, or nervous. Your body's response to adversity or demand is what we call stress. Among couples, male infertility is common. A failure in spermatogenesis is accountable for nearly half of all occurrences of infertility in marriage. Ageing, psychological stress, diet, physical exercise, coffee, hot water, hot scrotum, and cell phone usage are some of the few modifiable lifestyle variables that have a role in the development of infertility. Many hypotheses have been proposed to establish the link between stress in the workplace, life events (war, earthquake, etc.), and inability to conceive have been linked to inferior or degraded semen quality. In this review, we will discuss the effect on male fertility of elements including quality of life (such as exercise, diet, and other alterations to one's daily routine) and psychological stress. In addition, the effects on male fertility of elevated scrotal temperature, improper dietary habits, and physical inactivity will be discussed. The loss in male fertility, mainly due to ageing, inappropriate lifestyles, and environmental factors, is a significant public health concern in this century. Couples can enhance their quality of life and increase their chances of naturally conceiving a child by altering their way of life and supplementing it with nutraceutical antioxidants and an organised educational, environmental, dietary, and physical exercise program.

## Introduction and background

Fertility problems affect approximately 13% of the total reproductively active population, and male fertility issues are responsible for up to 30% of those cases [1]. Men's fertility may be decreasing because the quality of their semen has been shown to decline internationally [2-4]. Factors that explain this drop include a shift toward Western-style meals heavy in protein and lifestyle changes. There is an evolving panel of researchers investigating the link between sperm characteristics and lifestyle factors such as smoking [5], alcohol use [6], weight [7], physical activity [8,9], and food [10].

Drinks containing caffeine, such as coffee, and soda, contain 1,3,7-trimethylxanthine. Through various body fluids such as saliva, breast milk, embryo, and neonate [11], it has been discovered to be rapidly disseminated throughout the body. Caffeine is readily absorbed by humans, with a bioavailability of about 100% when given orally and a peak blood concentration of 15-45 minutes after ingestion [12]. The effects of caffeine include, but are not limited to, stimulation of the central nervous system, the elevation of catecholamine secretion, relaxation of muscle tissue, and stimulation of vital signs [13]. Both favourable and dire health consequences have been documented. Excessive consumption can harm health, including an increased risk for cardiovascular disease, hypertension, and neurological disorders [14]. Moderate consumption may protect against cardiovascular illnesses and glucose and lipid metabolism disorders. The rising popularity of caffeinated energy drinks, especially among teenagers, is cause for alarm [15].

Men's consumption of coffee or caffeine has been connected to elevated testosterone levels and the sex hormone binding globulin (SHBG) [16]. Coffee modifies the glycolytic and oxidative characteristics of the Sertoli Cells, which may interfere with a male's reproductive capacity [17]. There is, however, a lack of understanding of the mechanism by which caffeine may affect the body. When it comes to fetuses and adults, caffeine may have an altering effect on the hormonal system or directly on the germinative epithelium [17,18].

There has been a great deal of misunderstanding about how caffeine consumption impacts the general population in terms of infertility [19-26]. Coffee consumption may affect male reproductive function, according to a comprehensive study [19]. However, there has been no evidence of a link between caffeine consumption and male reproductive health. It is essential to establish a connection between coffee usage and infertility. As a result, we conducted a comprehensive analysis of all available clinical trial data to determine whether or not caffeine intake is linked to a higher likelihood of infertility in humans.

## Review

Methods

The terms like ‘coffee’, ‘caffeine’, ‘stress’, ‘male’, ‘fertility’, and ‘male infertility’ were searched for in a database like ‘PubMed’. Only results pertaining to the English language were shown. If there were more than one published report from a similar study, the latest one was used. Only review articles that also had original data were taken into account. We looked at all observational papers that focused exclusively on the link between a man's coffee or caffeine consumption and reproductive outcomes, such as sperm parameters, sperm DNA characteristics, and the ability to get pregnant.

Stress and infertility

Everyone experiences stress and infertility is no exception. This is because of the numerous social and medical pressures, as well as the associated tests, diagnoses, therapies, failures, disappointed hopes, and even financial expenditures [27]. LH and testosterone pulses can be affected by stress, and this, in turn, can alter spermatogenesis and the quality of sperm [28,29]. As evidenced by animal research, acute stress may interfere with testicular function. Germinal and Leydig cells perished in the testicular tissue of stressed rats [30,31]. Glucocorticoid receptors (GRs) have been found in Leydig, Sertoli, and germ cells [32,33]. However, the net consequences of stress may depend on how long it lasts. It is thought that high levels of glucocorticoid cause all cell types to die [31-33]. The main thing glucocorticoids do in the testes is controlled the Leydig cell. We do not know much about how glucocorticoids signal in the context of reproductive physiology right now. Stress has different effects on the brain, immune system, and behaviour in people. Recently, a single nucleotide NR3C1 polymorphism (BcII [rs41423247] [34]) was thought to show that the GR response to glucocorticoids affects how Sertoli and Leydig cells work. So, this variant gene is strongly linked to better sperm motility and testicular function [34]. This is because heterozygotes with this gene have an over-dominant form. The polymorphism of the GR in humans may indicate a range in how individuals respond to stress [35]. Sperm quality may be significantly negatively impacted by a solitary stressor, such as a job, life event, or even social strain, or by two concurrent stressful life events [27]. There was no change in volume or morphology, but there was a 39% decrease in sperm concentration, a 48% decrease in motility, and worse overall semen parameters on the day of oocyte retrieval, suggesting that the perceived stress of providing a semen sample was negatively linked to overall semen quality [36,37]. Additionally, important variables like environmental catastrophes, war, or ‘stressful life experiences’ cannot be quantified in terms of how they affect fertility, leading to an underestimating of the actual stress burden. Without the occurrence of particular stressful exposures, a high degree of stress can develop as a result of ongoing high stress in daily life. This could be the cause of the ambiguous findings, hence it is best to include a measure of perceived stress in any study that focuses on stress brought on by natural disasters or armed conflict [28,38-42].

Many studies show that the quality of sperm in men getting treatment for infertility or in men in the general population [43] decreases during infertility treatment. Still, these studies find it hard to tell whether anxiety is the source or the effect. Male impotence diagnoses, appointments, and failed IVF treatments can all lead to increased stress [27]. Most men undergoing infertility therapy were diagnosed with an anxiety disorder or depression [28]. Having to deal with different ways of living can also affect fertility. It was said that being assertive or aggressive as a way to deal with stress may negatively affect fertility by increasing adrenergic activity, which causes increased constriction of the blood vessels in the testes [44]. This narrowing of the blood vessels causes testosterone levels to drop and spermatogenesis to slow down. The association between anxiety and sexual tension was surprisingly high [45], even though men are not typically encouraged to talk about their anxiety or the sexual stress they experience. Research findings that examined men's self-reported levels of ‘daily-life stress’ while adjusting for relevant confounding factors showed conflicting results on sperm parameters. The researchers conducted both investigations on men from the general community. According to one study [46], perceived stress was found to have a negative relationship with sperm concentration, sperm motility, and the percentage of morphologically normal spermatozoa. Another study did not find any connection between the effects of stress and the qualities of the sperm. Still, it did indicate that a man's ability to produce children decreased in proportion to the stress he experienced [47]. Numerous studies have established a correlation between self-reported stress and low-quality sperm, posing a public health risk. Psychological stress may be an elastic or reversible element crucial in a clinical environment. Future research should attempt to scientifically quantify the impacts of stress and determine whether early counselling focused on reducing stress levels will improve sperm quality. They should also investigate the physiologic effects of stress on sperm quality.

Effect of caffeine on the fertility of males

Numerous beverages and foods contain caffeine, including tea, coffee, cola, energy drinks, and chocolate [48]. The impact of caffeine on one's health can be compared to a two-edged sword. It may protect against cardiovascular disorders (including coronary heart disease, arrhythmia, and heart failure), diabetes, liver disease [13], and possibly Parkinson's disease [14,49]. However, the association between caffeine consumption and semen parameters or male fertility has not been shown in the published literature. Determining the association between caffeine consumption and infertility is essential. To determine if caffeine use is a risk factor for infertility in humans, we conducted a systematic assessment of evidence from controlled clinical studies [49].

In Bugatti's study [50], men with azoospermia or oligospermia (i.e., sperms/mL 20,000,000,000) served as the case group. In contrast, males with normal regular examinations and sperm counts > 20,000,000,000/mL served as the control group throughout the same time frame. The study found no association between coffee consumption and sperm abnormalities (RR 0.91, 95% CI 0.46-1.82). Although it was stated that the coffee intake was separated into several doses for additional research, no relevant data were supplied, and the relative risk was not determined based on these doses. There were three groups in Prazzini's [49,51] study: infertile men with dyspermia (case group), normospermic infertile men, and fertile men with undetermined sperm quality (control groups). Our study included only the case group and the productive control group. The risk of dyspermia increased in direct proportion to the number of coffee beverages consumed each day. Those who consumed 200-300 mg/d of caffeine may have an OR of 1.7 (95%CI 0.8-3.7) compared to those who consumed less than 100 mg/d, and those who consumed more than 400 mg/d may have a higher risk of infertility (OR 3.4, 95% CI 2.4-12.6).

Relation Between Caffeine and DNA

Jurewicz et al. [52] and Robbins et al. [53] examined the association between lifestyle and sperm aneuploidy. In addition to aneuploidy, DNA integrity is an essential indicator of male infertility. The probable success of assisted reproductive technology (ART) by Radwan et al. [54] 140 failed to establish a correlation between DNA fragmentation as demonstrated by sperm chromosome structure assay and coffee use in a study of 290 healthy men [55]. Moderate consumption can not be associated with any investigated parameters of sperm DNA damage and DNA stainability. These parameters include the DNA fragmentation index (DFI), the medium DFI (M DFI), the high DFI (H DFI), and the high DNA stainability index (HDS - a fraction of immature sperms). By just using sperm Comet analyses, Schmid et al. [56] also aimed to investigate the relationship between coffee consumption and DNA damage in 80 healthy nonsmokers. They came to the conclusion that, regardless of age (older men have more sperm DNA damage), men who consume a lot of caffeine daily have increased sperm DNA damage linked to double-strand DNA breaks. Horak et al. [57] looked at the buildup of DNA adducts as a biomarker of exposure to chemical mutagens in a similar vein. A DNA adduct is a piece of DNA that has been chemically joined to a substance that causes cancer. It is possible that unrepaired DNA damage would accumulate during spermatogenesis due to DNA repair mechanisms that are stimulated by various chemicals and radiation occurring early during spermatogenesis but not in mature spermatids and spermatozoa [58]. The relationship between DNA adducts, fertility, and lifestyle choices has been studied, and among patients with impaired fertility, a substantial inverse relationship between the presence of DNA adducts and sperm concentration or motility was discovered. However, there was no link found between coffee consumption and sperm DNA adducts. In this regard, it should be taken into account that disagreements between research may be attributable to the diverse approaches to evaluating DNA damage. Sperm DNA fragmentation tests typically have a fair amount of concordance between them [59]. It is currently unclear which of these tests is better to facilitate clinical decision-making because none of them specifically reveal the type and extent of the DNA damage.

Relation Between Caffeine and ART

Klonoff-Cohen et al. [60] performed an investigation in existence to examine coffee consumers' success rate with ART. Despite the lack of specific outcomes, the researchers concluded that male caffeine use did not affect fertility, pregnancy, or live birth delivery. On the other hand, caffeine was identified as a risk factor for multiple pregnancies when examined as a continuous variable. A 100 mg/day increase in male caffeine use increased the likelihood of various gestations by 2.2 times (95% CI: 1.1-4.4) for lifetime consumption and by 3.0 times (95% CI: 1.2-7.4) for intake during the week of the initial clinic visit.

Relation Between Caffeine and Planned Pregnancy

The endpoint for five cohort studies was the time until a desired pregnancy [21,23,61-63]. Florack et al. [61] examined the relationship between caffeine intake (from coffee, tea, and cola) and feasibility in a prospective cohort of women working in non-medical functions at 39 Dutch hospitals and their partners. Men who consumed low or moderate amounts of caffeine did not vary, while those who consumed large amounts were more likely to experience a decline in fecundity. Males who drank four to seven caffeine drinks per day had an OR of 0.8 (95% CI 0.5 to 1.5), and those who drank eight or more had an OR of 0.6 (95% CI 0.3 to 0.97), compared to men who drank three or fewer caffeine drinks per day. Data from the Ontario Farm Family Health Study (retrospective cohort) were analysed by Curtis et al. [23] to determine whether consumption of caffeine, alcohol, and tobacco affected the fecundability ratio (FR), which is calculated by dividing the fecundability of the exposed group by that of the unexposed group. For this analysis, only intended pregnancies were chosen, accounting for 2,607 pregnancies among 1,277 couples. Coffee, tea, and beverages containing coke were all regarded as caffeine sources. When caffeine drinkers in men were compared to complete abstainers, a slight reduction in fecundity was discovered. However, because 96% of respondents admitted to consuming some caffeine, low (100 mg caffeine per day) versus high (>100 mg caffeine per day) consumption was also examined; no association with fecundity was found using this cut-point. There was no dose-response curve for caffeine use, and there were no interactions between caffeine use and cigarette smoking or between caffeine use in men and women. As a result, the study could not identify any link between overall caffeine use and fecundity. The consumption of each of the three drinks was examined as well. There was no overarching correlation between male coffee, tea, and cola consumers and fecundity. Consuming more than three cups of tea per day, however, was linked to lower fecundity (FR = 0.85, 95% CI 0.69-1.05), indicating that if the impact was real, it might have been brought on by ingredients other than caffeine. Jensen et al. [21] recruited 430 couples in Denmark who planned to stop using birth control to become parents but had no prior reproductive experience. Those union trade members between the ages of 20 and 35, who lived with a spouse and were childless, were enrolled as couples. The investigators looked at caffeine consumption across strata of smoking patterns and discovered no negative effects among male smokers. However, compared to nonsmokers who consumed 0-299 mg of caffeine daily, male nonsmokers with caffeine intake > 700 mg daily had a FR = 0.47 (95% CI 0.26-0.82). No statistically significant correlation between fecundability and consumption of any particular caffeine source was detected, however, both sources and total caffeine intake tended to have a similar effect. Cole et al. [62] goal were to retrospectively investigate the effects of maternal and paternal measures of persistent toxic substances on time to pregnancy among 41 couples from a general population, taking into account other known factors affecting fecundity when the woman was in the first trimester of pregnancy (the probability per month of becoming pregnant). For intake of more than 52 drinks each month (mean intake), the crude fecundability OR for paternal caffeine consumption was 0.49 (95% CI 0.20-1.20), and for couple consumption, it was 0.73. (95% CI 0.30-1.74). Couple caffeine consumption above the median as compared to below the median remained significantly linked (OR 0.25, 95% CI 0.10-0.63) with longer time to pregnancy in the multivariate model that included all relevant covariates but not male consumption.

In a North American prospective cohort study with 2,135 pregnancy planners, Wesselink et al. [63] investigated the relationship between preconception coffee intake and fecundability in male and female subjects. In this study, male caffeinated soda consumption had an inverse dose-response relationship with fecundability (1 and 2 vs. 0 cans/day: FR 0.77, 95% CI 0.56-1.05. And FR 0.72, 95% CI = 0.46-1.11). In contrast to caffeinated coffee, black tea, and green tea, male energy drink consumption was not linked with lower fecundability (1 vs 0 cans/day: FR 0.46, 95% CI 0.21- 0.98). While decaffeinated soda was not (>0 compared. 0 cans/day: FR 0.90, 95% CI 0.70-1.16), decaffeinated coffee (>0 vs. 0 cups/day: FR 0.73, 95% CI 0.46-1.17) and herbal/decaffeinated tea (1 vs. 0 cups/day: FR 0.64, 95% CI 0.32-1.31) were.

Outcome

Infertility in men was investigated in this research together with stress, coffee or caffeine use, and other lifestyle factors. Most studies did not find that drinking coffee, tea, or cocoa drinks with caffeine changed sperm parameters. On the other hand, some studies showed that cola drinks and soft drinks with caffeine were bad for volume, count, and concentration. When it came to DNA problems in sperm, caffeine seemed to be linked to aneuploidy and DNA breaks but not to other signs of DNA damage. Some studies, but not all, found that women who drank coffee took longer to get pregnant. Extremely different ways of measuring exposure, designing studies, and looking at the results of studies make it hard to say for sure what the link is between coffee intake and loss of male fertility. Especially meta-analyses cannot be done.

Coffee and other caffeinated drinks may be linked to strange eating habits or ways of living, and it is hard to tell the difference between spurious and potentially causal links. There may be a confounding effect in the relationship between different semen parameters and caffeine-containing soft drinks, but not the other way around. Soft drinks may therefore be more indicative than coffee. The links found for men who drank a lot of colas could not be explained by the amount of caffeine in cola, which was not very high. Instead, it may be that these men lead less healthy lives. Some caffeinated drinks could affect fertility in ways that have nothing to do with caffeine. For example, drinking cola-based drinks could lead to infertility by making you more likely to develop insulin resistance, metabolic syndrome, and gain weight [64-66]. Soda consumption by men in general, not just cola, has hurt the quality of their sperm [67]. As for the quality of the studies, most published articles that looked at the link between intake of caffeine and reproductive outcomes took into account potential or well-known confounding factors, such as the number of sexual partners, smoking, diseases of the reproductive organs, and alcohol consumption, age, and BMI. But it is not possible to rule out the effect of unmeasured confounders. Lastly, when looking at how long it takes to get pregnant or how often ART works, women's fertility is often overlooked, but it could be a very important factor. Studies on natural or ART-caused infertility can be affected by women-related confounders because both partners in a couple live at least some of the same way. In this complicated methodological situation, biological plausibility is a key part of guessing about possible causes, which needs some discussion. So, due to the lack of clear criteria for evaluating the calibre of semen research until recently, inaccurate conclusions may have been drawn as a result of skewed data from earlier studies with no quality controls, which may have contributed to the observed heterogeneity.

**Figure 1:**
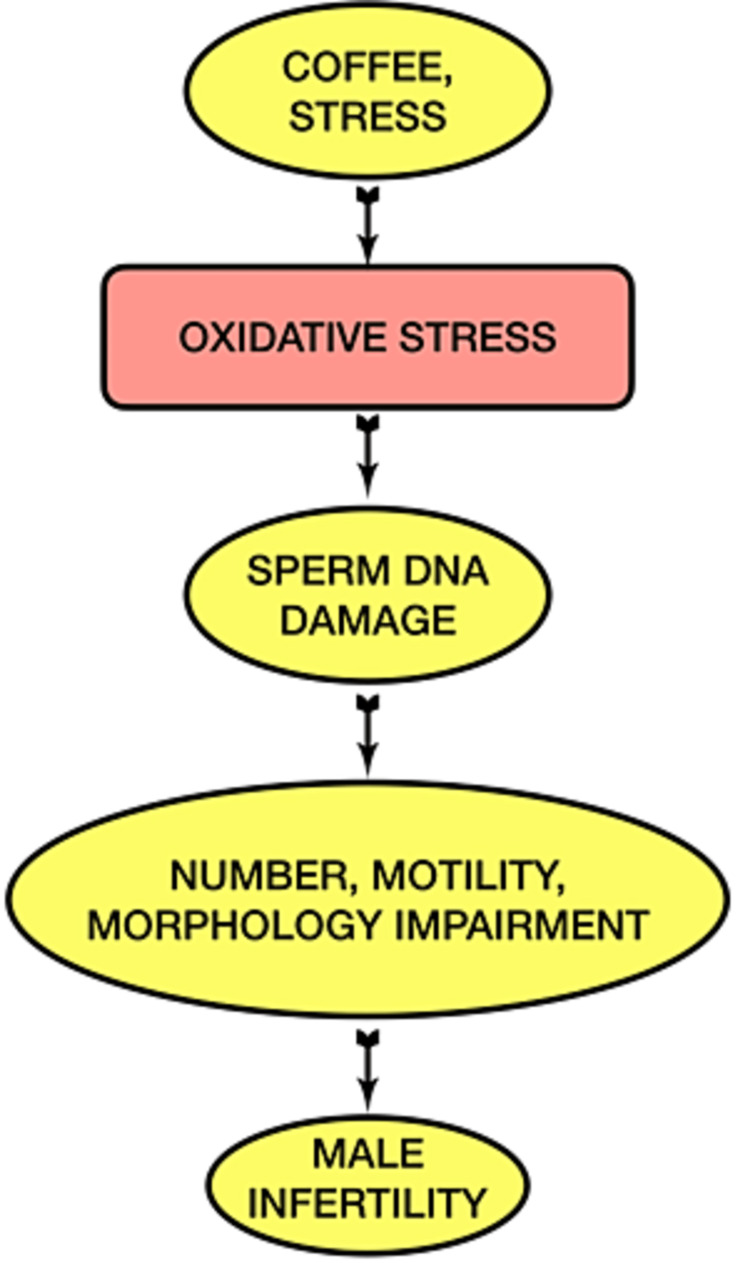
Schematic representation of the effect of caffeine and stress on male fertility This illustration has been created by the author (Mayank Kumar) of this study.

Because of the significance of lifestyle variables in forming male infertility, many people are becoming interested in this topic (Figure 1) . There is a correlation between one's mental attitude and their ability to have children. Still, at the moment, it is hard to prove that one causes the other. The stress of any kind (physical, emotional, biological, etc.) can lower a man's ability to have children, but there is no general agreement on how to measure it objectively. We feel comfortable recommending that both partners' ages be considered to increase the possibility of conception and lower the risk that the baby will have a genetic disorder. Based on this evidence, we recommend being careful when talking to older couples who want to use ART to get pregnant. They should be reassured as soon as possible to reduce their exposure to stressors. Several studies have shown that antioxidants can help reverse sperm dysfunction caused by oxidative stress in patients with idiopathic male infertility [68]. You should think about using them, but you should not do so before getting a diagnosis because it's hard to find solid evidence on this subject. Both pre-clinical and clinical studies found that bad eating habits seemed to make sperm parameters worse. Men may be more fertile if they get the right nutrients and lose weight over time. Men who have abnormal BMI or suffer from compulsive eating are more prone to have hormonal problems that can make them less able to have children. So far, only cross-sectional or case-control studies have given us information about how to choose supplements or food groups [69]. We do not have conclusive information about them, like caffeine. It's important to promote access to andrologists so that they may offer lifestyle advice and potentially prescribe dietary antioxidants. The correction of improper lifestyle could reduce the scope of DNA fragmentation and make it easier to provide better quality of life for people who are trying to conceive [54].

## Conclusions

Evidence that has been made public suggests that drinking coffee may hurt a man's ability to have children, possibly because it damages the DNA of sperm. But we should be cautious with this conclusion. To get a good idea of how caffeine affects sperm parameters and male fertility, you need well-planned studies with clear criteria for analysing sperm and choosing subjects and a clear idea of how people live their lives. We think that all of the observational studies that have been used to talk about these nutritional factors may show associations but not causes. To confirm these correlations, we need well-designed randomised controlled trials which for ethical issues cannot be done on humans.
